# Supplementary material for: The Clinical Effect of a Propolis and Mangosteen Extract Complex in Subjects with Gingivitis: A Randomized, Double-Blind, and Placebo-Controlled Clinical Trial
Source: Nutrients. 2024 Sep 5;16(17):3000. doi: 10.3390/nu16173000 (PMC11396876; doi:10.3390/nu16173000)
Supplement: Supplementary file 1 [file nutrients-16-03000-s001.zip › nutrients-3171162-supplementary.pdf]

NS NUTRATECH AUSTRALIA

26/2 Slough Avenue, Silverwater NSW 2128 Australia
Phone : +61 433 030 115 Fax: +61 2 9411 7923

CERTIFICATE OF ANALYSIS

Product Name : Propolis Powder

Batch No. : PW-PP-70-202006a	
Appearance : Brown Fine Powder	Storage : Avoid sunlight & keep cool and dry
Package : 5kg / Bags, 4bags / Carton	Manufacture Date : 2020-06-25
Shelf Life : 60 Months	Origin : Australia

Ingredients	
Ethanol – Extracted Propolis Powder	70%
Malto - Dextrin	30%

Ingredient Test	Specification	Result
Propolis Content	≥ 70%	70.8%, complies
Flavonoids Content	≥ 9%	9.8%, complies
Moisture	≤ 5%	1.9%, complies
Diethylene Glycol Content	Not detected	Not detected, complies
p-Coumaric Acid	Detected	Detected, complies
Cinnamic Acid	Detected	Detected, complies
Tetracycline	Not detected	Not detected, complies
Chlortetracycline	Not detected	Not detected, complies
Microbial Release Limits: BP**	Specification	Result
Total Viable Aerobic count (TVAC)	NMT 10,000 cfu/g	<10 cfu/g, complies
Total Yeast and Mould	NMT 100 cfu/g	<10 cfu/g, complies
Bile-tolerant Gram negative bacteria	NMT 100 cfu/g	<10 cfu/g, complies
Escherichia Coli	Not detected in 1 gram	Not detected/g, complies
Coliform Group	Negative	Negative, complies
Salmonellae	Not detected in 10 gram	Not detected/10g, complies
Staphyloccoccus Aureus	Not detected in 1 gram	Not detected/g, complies
Heavy Metal Tests : BP**	Specification	Result
Pb	NMT 1.0 ppm	0.30 ppm. complies
As	NMT 0.5 ppm	0.12 ppm, complies
Hg	NMT 0.04 ppm	0.02 ppm, complies
Cd	NMT 0.05 ppm	0.01 ppm. complies

*Material has been quantified by input
** All references to the BP are the current edition. This batch has underwent microbial analysis

Issued and Approved by:

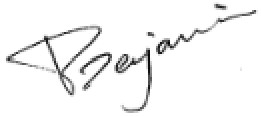

Benjamin Anderson
Director
Date: 20-01-2021

SAVA HEALTH CARE LIMITED
Plot No. 107,108,109, Phase III,
Kiadb Industrial Area
Malur 563160
Karnataka, India
Tel : +912030516100

Certificate of Analysis

Product : **Mangosteen Rind Extract Powder**

Customer Name :	Taesung Co Ltd	Batch No. :	MGE 2024 / 002 S
Product Code :	GME 40	Mfg. Date :	February 19, 2024
Source :	Garcinia Mangostana	Expiry Date :	February 18, 2027
Quantity :	100g		

Parametres	Specification	Result
Description	Yellowish brown to brown powder	Complies
Odour & Taste	Characteristic	Complies
Loss on drying	NMT 5.0 %	3.52%
Bulk Density (Tapped)	0.3 – 0.80 g/ml	0.74 gm/ml
Particle Size	100% passing through 40 mesh	100%
Identification	Presence of Mangostin	Complies
**Assay**		
Alpha Mangostin	NLT 40.0 %	42.88 %
Microbiology Test		
Total Plate Count	NMT 3,000 cfu / g	Complies
Yeast & Mold	NMT 100 cfu / g	Complies
Coliforms	Absent	Absent
E.Coli	Absent	Absent
Salmonella	Absent	Absent
**Heavy Metals**		
Lead	NMT 2 ppm	Complies
Arsenic	NMT 1 ppm	Complies
Cadmium	NMT 0.5 ppm	Complies
Mercury	NMT 0.1 ppm	Complies
GMO Free	Complies	Complies
Halal & Kosher	Certified	Complies
GMO, BSE/TSE, Allergen	Free	Complies

The product complies with the above specification.

Since it is an herbal Product, there is likely to be minor colour variation from batch to batch because of the seasonal variations of the raw materials. Colour change will not affect the quality of the product. To be kept in a cool and dry place away from light.

Analysed by	Reviewed by	Approved by
QC Chemist	QC Executive	QC Manager

Certificate of Analysis

Product Name	Lactose95 (100M)	Date of manufacture	2024-05-07
Type of food	Other processed products	Best before date	2025-04-16
Dimensions	25Kg	Packaging method	25Kg
Account Manager	Kwang Eon Kim		

The test results of this item are as follows

Results of the analysis of Management standard and product analysis results

Classification	Analyzed items	Inspection standard	Analysis method	Analysis completion date	Analysis result	Experimenter	Judgment
Self-test	Appearance	The product has its own color and flavor and should be free of off-flavors.	Organoleptic test	2024-05-17	Passed	Kwang Eon Kim	Passed
	foreign substance	not detected	visual inspection	2024-05-17	not detected	Kwang Eon Kim	Passed
Inspected by an accredited laboratory	foreign substance	not detected	food code test	2023-10-17	not detected	BioFood Lab	Passed

- Food type: Other processed products
- Ingredients: Lactose 95% (USA), dextrin
- Expiration date: 11 months from the date of manufacture

Rich Meal 2nd Factory

Address: 70-1, Inhwa-gil, Jincheon-gun, Chungcheongbuk-do, Korea

TEL:(043)532-5400 FAX:(043)537-3378

Translated the following document, originally written in Korean by Dr. Suk Ji, ____________

August 19, 2024

RICH MEAL

시험성적서

제품명	유당95(100M)	제조일자	2024-05-17
식품의유형	기타가공품	소비기한	2025-04-16
규격	25kg	포장방법	25kg
담당자	김 광 언		

해당 품목 시험 결과는 다음과 같습니다.

관리규격 및 제품분석 결과

구분	분석항목	검사규격	분석방법	분석완료일	분석결과	실험자	판 정
자체검사	성상	고유의 색택 및 향미를 가지고 있으며 이미,이취가 없어야 한다	관능검사	2024-05-17	적합	김광언	적 합
	이물	불검출	육안검사	2024-05-17	불검출	김광언	적 합
공인기관 검사	이물	불검출	식품공전에 따름	2023-10-17	불검출	바이오 푸드랩	적 합

- 식품유형 : 기타가공품
- 원재료명 : 유당95%(미국산), 덱스트린
- 소비기한 : 제조일로부터 11개월까지

리치밀 제2공장

주소:충북 진천군 덕산읍 인화길 70-1

TEL:(043)532-5400 FAX:(043)537-3378

AMSTER®

QUALITY CONTROL
CERTIFICATE OF ANALYSIS

Product: MICROCRYSTALLINE CELLULOSE E460 (i)
Grade : AMCELL PLUS 102
Batch No.: A24030133
Batch Size: 3.000 MT
Mfg. Date: Mar'2024
Re-evaluation Date: Feb' 2029

**Description**	White or almost white fine or granular powder.
**Solubility**	Practically insoluble in water, dilute acid and most of organic solvents, Slightly soluble in dilute NaOH solution

Pharmacopeial test items	Specification	Results
Identification		
Solubility	Have to correspond as E460(i)	Complies
Colour reaction	Have to correspond as E460(i)	Complies
Infrared absorption spectroscopy	Have to correspond as E460(i)	Complies
Suspension test	Have to correspond as E460(i)	Complies
pH	5.0-7.5 as E460(i)	6.53
Water - Soluble Substance	NMT 0.24% as E460(i)	0.1500%
Loss on Drying	NMT 5.0% as E460(i)	3.8500%
Sulphated Ash	NMT 0.5% as E460(i)	0.0490%
Starch	Not detectable as E460(i)	Complies
Carboxyl group	NMT 1% as E460(i)	Complies
Lead	NMT 2.0 mg/kg as E460(i)	Complies
Arsenic	NMT 3.0 mg/kg as E460(i)	Complies
Mercury	NMT 1.0 mg/kg as E460(i)	Complies
Cadmium	NMT 1.0 mg/kg as E460(i)	Complies

In house Standards	In house Specification	
Bulk Density	0.27 to 0.34 g/ml	0.3140 g/ml
Assay (Dried)	97.0% to 102.0%	99.50 %
Sieve Analysis (% Retention)		
60 Mesh	≤ 8.0%	0.56 %
200 Mesh	≥ 45.0%	55.28 %

MICROBIAL LIMITS

Total Viable Aerobic Count	N.M.T. 1000 cfu/g as USP/NF, JP, Ph.Eur., BP	40
Total yeast & Mold Count	N.M.T 100 cfu/g as USP/NF, JP, Ph.Eur., BP	< 10
Staphylococcus aureus	Absent as USP/NF, JP, Ph.Eur., BP	Absent
Escherichia Coli.	Absent as USP/NF, JP, Ph.Eur., BP	Absent
Pseudomonas aeruginosa	Absent as USP/NF, JP, Ph.Eur., BP	Absent
Salmonella species	Absent in 10g as USP/NF, JP, Ph.Eur., BP	Absent

The raw materials, manufacturing process, and product do not contain any of solvents listed in Organic Volatile Impurities (USP<467>) & residual solvents (Ph – Eur<5.4>)

Storage recommendation: Preserve in tight containers.

Lalit Patel
Quality control

# TO WHOM SO EVER IT MAY CONCERN

GMO CERTIFICATE

We AMSTER MICROCELL PVT. LIMITED certify that to the best of our knowledge, Microcrystalline cellulose does not contain any material derived from genetic modification (GM). Additionally, this product has not come into contact with any GM materials during its manufacturing, packing or storage.

For, Amster Microcell Pvt. Ltd.

Jayesh Prajapati
QA/QC Manager
Date: 03-10-2020

AMSTER MICROCELL PVT. LTD. * AMSTER MICROCELL PVT. LTD.

AMSTER MICROCELL PVT. LTD.
Survey No. 131, Chadasna, Nr. D.K. Industrial Estate, Behind Chhatral GIDC Phase III,
Chhatral - Ambapura Road, Tal. Kadi, Dist. Mehsana - 384450.
www.amstermicrocell.com
CIN : U24304GJ2018PTC101080

MITSUBISHI CHEMICAL GROUP

MITSUBISHI CHEMICAL CORPORATION
Palace Building, 1-1, Marunouchi 1-chome,
Chiyoda-ku, Tokyo 100-8251, Japan
Phone : +81-3-6748-7425 Facsimile : +81-3-6685-5465

February 06, 2024

To : Nam Yung Commercial Co., Ltd.

CERTIFICATE OF ANALYSIS
of Ryoto Sugar Ester P-1670

Name	Sucrose esters of fatty acids
Product Name (Used on Label)	RYOTO SUGAR ESTER P-1670
Lot number	39111211
Quantity	7800kg
Manufacturing date	September 11, 2023
Best before date	September 10, 2025

	Items	Specifications	Results
1	Appearance	White to yellowish brown powder	pass
2	Identification (1)	Specimen shall pass the test.	pass
3	Identification (2)	Specimen shall pass the test.	pass
4	Acid value	Not more than 5.0 *	1.0
5	Lead	Not more than 1.0 μg/g *	pass
6	Arsenic (as As)	Not more than 0.75 μg/g *	pass
7	Cadmium **	Not more than 1.0 μg/g	pass
8	Mercury **	Not more than 1.0 μg/g	pass
9	Free sucrose	Not more than 5.0%	0.4%
10	Dimethyl sulfoxide	Not more than 2.0 μg/g	pass
11	Dimethylformamide	Not more than 1.0 μg/g	pass
12	Ethyl methyl ketone	Not more than 10 μg/g	pass
13	EA, IPA and PG ***	Not more than 0.035% (in combination)	pass
14	Methanol	Not more than 10 μg/g	pass
15	2-Methyl-1-propanol	Not more than 10 μg/g	pass
16	Water content	Not more than 4.0%	0.7%
17	Residue on ignition	Not more than 1.5% *	0.2%

* : Lower than that defined in Japan's Specification and Standard for Food Additives (9th Edition)
** : Not defined in Japan's Specification and Standards for Food Additives (9th Edition)
*** : EA : Ethyl acetate, IPA : 2-Propanol, PG : Propylene glycol

Sucrose esters of fatty acids is approved as a food additive in Japan under the Food Sanitation Law.

T. Fukazawa

Tetsuya Fukazawa
Group Manager
Foods QA Group, Food/Healthcare Department

RT80 카라멜색소

SETHNESS ROQUETTE

EXCELLENCE IN CARAMELS

CERTIFICATE OF ANALYSIS

RT80-05EX Class II Liquid Caramel Color   Lot Number: LD030005EX

Date Manufactured: June 08, 2020   Best Used by: June 08, 2022

Test Description	Result	Low Spec	Hi Spec	Method
Tinctorial Power, K0.56 *	0.091	0.082	0.092	SETHNESS
Color Intensity (typical) **	0.045	0.039	0.048	SETHNESS
Hue Index,s (typical, no spec)	6.31	5.80	6.60	SETHNESS
BAUME' @60F, hydrometer	38.70	38.30	38.70	AOAC 932.14A
Specific Gravity @60F, calc.	1.364	1.359	1.364	AOAC 932.14B
pH, "as is"	2.96	2.90	3.30	AOAC 945.10
Ionic Character	NEGATIVE ***			
Lead (Pb), ppm	< 2 ***	0.000	2.000	ICP
Arsenic (As), ppm	< 1 ***	0.000	1.000	ICP
Mercury (Hg), ppm	< 0.1 ***	0.00	0.10	ICP
Cadmium (Cd), ppm	< 0.03 ***	0	1	ICP
Sulfur Dioxide, ppm /0.10 CI	950 ***	0	2000	FCC 6th Ed.
Total Nitrogen, %/0.10 CI	0.23 ***	0.0	3.3	FCC 6th Ed.
Total Sulfur, %/0.10 CI	3.09 ***	0.0	3.5	FCC 6th Ed.

* 0.1% solution absorbance/cm @ 560 nm
** 0.1% solution absorbance/cm @ 610 nm
*** on an audit basis

The above caramel color complies with the color additive regulation defined under CFR Title 21 Sec. 73.85.
Country of Origin: United States of America

CONTAINS SULFITES. SEE NUTRITIONAL STATEMENT FOR SPECIFIC SULFITE INFORMATION.

Jason M. Ernst   07/15/2020

Jason M. Ernst   Date

Manufactured by: SETHNESS PRODUCTS CO.   CLINTON IA. 52732

TO: JIN FOOD
ATTN: MS. HANA CHOI
6FL O.S.L. B/D,
316-9 YANGJAE-DONG SEOCHO-KU

SEOUL 137-130
SOUTH KOREA

Customer: 39279
Order #: 484431
PO Number: JI-11860
Product Code:

SETHNESS PRODUCTS COMPANY
1347 BEAVER CHANNEL PARKWAY
CLINTON, IOWA 52732
PHONE (563) 243-3943 FAX (563) 243-1663

PQ
PT. PQ Silicas Indonesia

Jl. Rembang Industri Raya No 24 Pasuruan 67152
Pasuruan Industrial Estate Rembang (PIER), East Java – Indonesia
Phone: (62) (343) 740 111
Fax: (62) (343) 740 113
www.pq.com

CERTIFICATE OF ANALYSIS

22th May 2024

TAE WANG MULSAN CO., LTD.
111 BANGBAE - RO
SEOCHO – GU
SEOUL 06683
KOREA

Commodity: Silicon Dioxide Neosyl GP Food Grade Precipitated

Production date 08.05.24
Expire date 08.05.26

Batch No. 4194

Standard Method of Analysis	Parameter	Specification	Result	Units
IK.QCD.LAB.16b	Average Particle Size (Malvern 3000)	18.0 max	15.6	µm
IK.QCD.LAB.13	Loss at 1000°C (wet basis)	12.0 max	8.8	%
IK.QCD.LAB.14	pH (5% aqueous suspension)	6.0 – 8.0	6.9	
IK.QCD.LAB.12	Oil Absorption (Linseed)	200 – 330	274	g/100g
IK.QCD.LAB.17	Colour – Harrison	96 min	98	
IK.QCD.LAB.15	Sodium Sulphate	5 max	1.0	%
SAM 024.04B	Assay, Silica*	96.0 min	98.6	%
LSAM-TM01a	Arsenic as As*	2 max	<0.25	ppm
LSAM-TM01a	Lead as Pb*	5 max	0.3	ppm
LSAM-TM01a	Cadmium as Cd*	2 max	<0.06	ppm
LSAM-TM20a	Heavy metals*	15 max	1.2	ppm

**Typical Data*

Pasuruan, (DD/MM/YY) : 22/05/24

MUHAJIR
QA Manager

SENSIENT® **Sensient Certificate of Analysis**

Date: 26-May-2023
Time: 19:47:06

Customer PO# 320566
Customer Item# 9180105
Customer Description FD&C BLUE NO. 1 POWDER 20KG
Sales Order# 23009555

Customer Name BOLAK COMPANY LTD
Customer Address Room 302, Bukchang Blgd., 20-6
Bukchang-Dong
Choong-Ku
100- 080 Seoul

Sensient Item# 056011321
Sensient Description FD&C BLUE NO. 1 POWDER
Lot# BC3433
Manufacture Date 19-Mar-2023
Best Before Date 17-Mar-2029

Manufactured location: 2526 Baldwin Street, St. Louis MO 63106

PD% 91

Test Description	Min Value	Max Value	Test Value	Test Method
ARSENIC PPM	0	1.00	0.01	
CHROMIUM PPM	0	50.00	0.63	
Manganese	0	50.00	28.61	
HEAVY METALS PPM	0	10.00	6.72	
%TOTAL SODIUM CHLORIDE/SULFATE	0	4.00	2.64	

This color is manufactured to comply with:
US Code of Federal Regulations, Title 21, Parts 73 and 82.
Regulation (EC) No 231/2012 laying down specifications for food additives.
FAO/WHO JECFA Specification - Online Edition.
The Japanese Standard for Food Additives.
The Korean Food Additives Code current edition.
Lead--<2 ppm
Arsenic--<1 ppm
Mercury--<1 ppm
Cadmium--<1 ppm
Unsulphonated Primary Aromatic Amines (as Aniline) --<100 ppm or 0.01%
Ether Extracts--<0.2%
Heavy Metals--<20 ppm
Insoluble Matter--<0.2%
Iron--<100 ppm
This product complies with the purity guidelines as outlined in
GB 1886.217-2016 for Brilliant Blue.
Chromium <50 ppm.

Francois Henning, QC Manager

Confidential: This transmission is intended for the use of the named addressee(s). If you have received this transmission in error, please destroy all copies and notify us immediately.

LOT NO: BC3433 FD&C BLUE No. 1

Sensient Colors LLC

Batch No.: 5883876 Weight: 4,761.94 LBS APR-10-2023

DETERMINATION	METHOD	FOUND	UNIT	DATE
TARTRATE BUFFER TITRATION	1	91.2	%	APR-04-2023
SPECTROPHOTOMETRIC	2	90.56	%	APR-06-2023
VOLATILE MATTER	3	3.3	%	APR-03-2023
NACL	4	1.26	%	APR-10-2023
NA2SO4 BY I. C.	5	1.16	%	APR-10-2023
WATER INSOLUBLE MATTER	6	0.02	%	APR-04-2023
LEUCO	56	2.45	%	APR-06-2023
SULFOBENZALDEHYDE (O,M,P)	58	<0.02	%	APR-06-2023
ESBSA	59	0.05	%	APR-06-2023
LOWER SULFONATED SUBSIDIARY	60	2.11	%	APR-06-2023
MERCURY	7	PT	ppm	APR-05-2023
LEAD	8	<2	ppm	APR-05-2023
ARSENIC	9	<1	ppm	APR-05-2023
MANGANESE	196	28.61	ppm	APR-05-2023
CHROMIUM	197	<2	ppm	APR-05-2023

CALLUP DUPLICATE - NOT OFFICIAL COPY
Food and Drug Administration Analytical Results
